# Neuroanatomical and cellular degeneration associated with a social disorder characterized by new ritualistic belief systems in a TDP-C patient vs. a Pick patient

**DOI:** 10.3389/fneur.2023.1245886

**Published:** 2023-10-11

**Authors:** Daniel T. Ohm, Emma Rhodes, Alejandra Bahena, Noah Capp, MaKayla Lowe, Philip Sabatini, Winifred Trotman, Christopher A. Olm, Jeffrey Phillips, Karthik Prabhakaran, Katya Rascovsky, Lauren Massimo, Corey McMillan, James Gee, M. Dylan Tisdall, Paul A. Yushkevich, Edward B. Lee, Murray Grossman, David J. Irwin

**Affiliations:** ^1^Penn Digital Neuropathology Laboratory, Department of Neurology, University of Pennsylvania, Philadelphia, PA, United States; ^2^Penn Frontotemporal Degeneration Center, Department of Neurology, University of Pennsylvania, Philadelphia, PA, United States; ^3^Penn Image Computing and Science Lab, Department of Radiology, University of Pennsylvania, Philadelphia, PA, United States; ^4^Center for Advanced Magnetic Resonance Imaging and Spectroscopy, Department of Radiology, University of Pennsylvania, Philadelphia, PA, United States; ^5^Center for Neurodegenerative Disease Research, Department of Pathology and Laboratory Medicine, University of Pennsylvania, Philadelphia, PA, United States

**Keywords:** behavioral variant frontotemporal dementia, primary progressive aphasia, Pick disease, TDP-C, semantic knowledge, social cognition, anterior temporal lobe, cortical layers

## Abstract

Frontotemporal dementia (FTD) is a spectrum of clinically and pathologically heterogenous neurodegenerative dementias. Clinical and anatomical variants of FTD have been described and associated with underlying frontotemporal lobar degeneration (FTLD) pathology, including tauopathies (FTLD-tau) or TDP-43 proteinopathies (FTLD-TDP). FTD patients with predominant degeneration of anterior temporal cortices often develop a language disorder of semantic knowledge loss and/or a social disorder often characterized by compulsive rituals and belief systems corresponding to predominant left or right hemisphere involvement, respectively. The neural substrates of these complex social disorders remain unclear. Here, we present a comparative imaging and postmortem study of two patients, one with FTLD-TDP (subtype C) and one with FTLD-tau (subtype Pick disease), who both developed new rigid belief systems. The FTLD-TDP patient developed a complex set of values centered on positivity and associated with specific physical and behavioral features of pigs, while the FTLD-tau patient developed compulsive, goal-directed behaviors related to general themes of positivity and spirituality. Neuroimaging showed left-predominant temporal atrophy in the FTLD-TDP patient and right-predominant frontotemporal atrophy in the FTLD-tau patient. Consistent with antemortem cortical atrophy, histopathologic examinations revealed severe loss of neurons and myelin predominantly in the anterior temporal lobes of both patients, but the FTLD-tau patient showed more bilateral, dorsolateral involvement featuring greater pathology and loss of projection neurons and deep white matter. These findings highlight that the regions within and connected to anterior temporal lobes may have differential vulnerability to distinct FTLD proteinopathies and serve important roles in human belief systems.

## Introduction

The neurobiology of cognitive and social impairments in the clinically and pathologically heterogeneous spectrum of frontotemporal dementia (FTD) syndromes is poorly understood (1). The most prominent symptoms of FTD include socio-emotional, language, and motor impairments that occur in various combinations and severity (2). Moreover, the neuropathological substrates for FTD include various subtypes of frontotemporal lobar degeneration (FTLD) proteinopathies, most commonly TDP-43 proteinopathies (FTLD-TDP) and tauopathies (FTLD-tau) (3). FTLD-TDP and FTLD-tau have a similar probability of causing the behavioral variant of FTD (bvFTD), the most common FTD syndrome (4–6). In contrast, variants of primary progressive aphasia (PPA) have some predictive value for specific forms of FTLD (7–11); However, these associations are not absolute, highlighting the clinicopathologic heterogeneity of FTD.

Anatomical patterns of degeneration may help explain clinical and pathologic heterogeneity in FTD (12–14). Neuroimaging studies suggest temporal variants of FTD exhibit early atrophy of the anterior temporal lobes (aTL) linked to either a bvFTD-like syndrome or the semantic variant of PPA (svPPA) depending on the predominant hemisphere involved (15–17). For example, the left temporal variant is most often associated with FTLD-TDP subtype C (TDP-C) pathology and symptoms characteristic of svPPA such as anomia and semantic knowledge loss (15, 18–20). In contrast, the right temporal variant is associated with heterogenous underlying FTLD pathologies and various behavioral and cognitive features including rigid compulsions, goal-oriented rituals, disinhibition, empathy loss, episodic memory loss, topographagnosia, prosopagnosia, depression, somatic complaints, and non-verbal semantic impairments (e.g., biographical person-specific knowledge) (21–30). Moreover, left and right temporal variants of FTD often progress into mixed clinical phenotypes that share similar language and behavioral symptoms within 3–5 years of symptom onset (15, 16, 31), suggesting a clinicoanatomic spectrum of svPPA and bvFTD (30). Interestingly, there also can be an emergence of new artistic creativity and/or new values/beliefs in FTD patients, especially those with predominantly temporal lobe disease (32–34), but the cellular and pathologic correlates of these behavioral symptoms are understudied.

Here, we compare detailed antemortem and postmortem data from one patient with FTLD-tau (Pick disease, PiD) and one patient with FTLD-TDP-C, both of whom developed semantic impairments and social disorders that included complex ritualistic behaviors that manifested as novel and concrete belief systems, to uncover shared and disparate cellular contributions to clinical symptoms in FTD.

## Methods

Patients were enrolled in observational research at the Penn Frontotemporal Degeneration Center (FTDC). Clinical diagnoses were established at FTDC consensus meetings using clinical criteria (6, 7). Neuropathologic diagnoses were performed using established methods and current neuropathologic diagnostic criteria (35–37). Patients were genotyped for FTD related genes and were negative for pathogenic mutations in *C9orf72*, *GRN*, *MAPT*, *VCP* and > 20 other genes associated with neurodegenerative disease as described previously (38). Please see Supplementary material for magnetic resonance imaging (MRI) and digital histopathology methods.

## Results

### FTLD-TDP patient

The first patient was a 67-year-old, right-handed White man with 12 years of education who presented to the Penn FTDC with insidious onset of language problems for 2 years. The patient reported difficulties with naming, understanding the meaning of common words (e.g., deer, acorn), and difficulty spelling, while memory for recent events and conversations was not reported to be problematic. These language changes were accompanied by subtle changes in personality and behavior characterized by reduced social inhibitions (e.g., inappropriate comments about a stranger’s body), dysphoria (e.g., crying more often), impatience with his family, and hyperorality for sweets.

On baseline examination, his speech was fluent but featured word-finding pauses, circumlocutions, semantic paraphasic errors (e.g., subordinate substitutions), and semantic knowledge loss (e.g., described a camel drawing as a “European domesticated animal”). He demonstrated worse semantic- than letter-fluency performance and had surface dyslexia. Basic and complex comprehension was intact, and he wrote a complete sentence. He demonstrated retrieval-based verbal memory deficits with relative preservation of visual memory. Set-shifting was weak, but there was no evidence of visuospatial impairment, apraxia, or acalculia. His Mini-Mental State Examination (MMSE) was 24/30, indicating mild impairment. Elemental neurologic examination was unrevealing. At initial visit, he met clinical diagnostic criteria for svPPA, and neuropsychological testing showed relatively isolated semantic loss (Supplementary Table 1).

**Figure 1:**
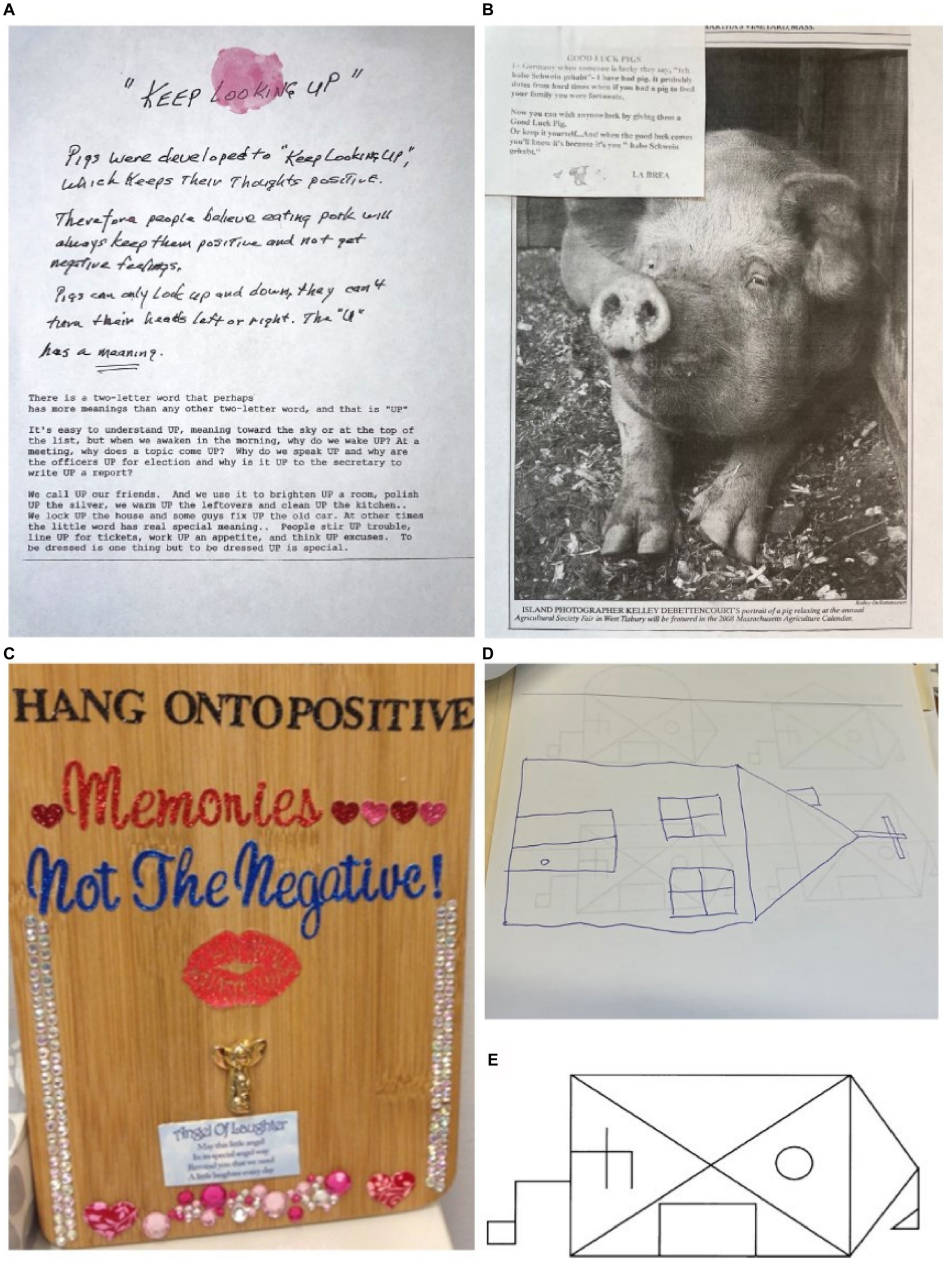
Personal items and visual reproductions illustrating evidence of new and rigid belief systems and ritualistic compulsions in both patients. **(A)** A written explication of the FTLD-TDP patient’s personal mantra, “keep looking up,” which derives positive emotional meaning from the pig’s physical characteristic of only being able to look up and down rather than side to side. His belief system featured semantically rich associations of complex and abstract concepts (e.g., intelligence, creativity) with concrete, physical characteristics. **(B)** A sampling of images and written material pertaining to pigs collected by the FTLD-TDP patient, including a German fable and a photo of a pig from an agricultural fair. The fable is titled “GOOD LUCK PIGS” that FIGURE 1 (Continued)reads, “In Germany when someone is lucky they say ‘Ich habe Schwein gehabt’—I have had pig. It probably dates from a hard time when if you had a pig to feed your family you were fortunate. Now you can wish anyone lucky by giving them a Good Luck Pig. Or keep it yourself… And when the good luck comes you’ll know it’s because it’s you [*sic*] ‘habe Schwein gehabt.’” He became obsessive with collecting images and figurines of pigs, and often gave these out to people when discussing his belief system. **(C)** A cutting board that the FTLD-tau patient decorated with messages of positivity, including instructions to “hang onto positive memories not the negative!” and a poem titled “Angel of Laughter” that reads, “May this little angel/In its special angel way/Remind you that we need/A little laughter every day.” Her obsessive fixation on positivity was accompanied by compulsive production of positivity-themed visual arts and crafts featuring religious and spiritual iconography. **(D)** Delayed recall trial of the Benson complex figure test showing evidence of enhanced visual artistic creativity despite impaired memory for the target stimulus in the FTLD-tau patient. She continued to ritualistically produce arts and crafts as her disease progressed, but her work became less focused on positivity and reflected semantic knowledge loss (e.g., drawing a turkey with tentacles instead of legs). **(E)** The original Benson complex figure stimulus for reference to the delayed recall copy pictured above.

Within 4 years, a more prominent social disorder emerged featuring a new fixation on pigs, complex goal-directed ritualistic behaviors, and worsening disinhibition (e.g., offering money and food to unfamiliar people/children). His fixation on pigs centered around positive qualities he attributed to them (e.g., intelligence, sensitivity; Figure 1A) and physical characteristics he interpreted as meaningful (Figure 1B). “Keep looking up” became a personal mantra that he readily shared with strangers, and he no longer showed signs of depressed mood. He became increasingly rigid, adhering to a strict exercise routine and hyper-oral diet (i.e., restricted to hamburgers, hot dogs, and cookies eaten in a ritualized manner). Other compulsive behaviors included counting and a specific cleaning routine, with significant distress if disrupted. His speech became more tangential, and he expressed a wish that he could communicate in numbers instead of words at a late-stage visit. Empathy further reduced, and while frustrated and concerned by his language impairments, he had limited insight into his behavioral changes.

His longitudinal cognitive profile (Supplementary Table 1) reflected progressive semantic knowledge loss and eventually impaired episodic memory and attention, with mild global cognitive impairment (CDR = 1, MMSE = 23, 8 years after onset). He became more globally and functionally impaired (CDR = 2, MMSE = 19) 12 years after onset and died with end-stage dementia after 15 years.

Neuropathologic examination found TDP-43 pathology in the form of mostly dystrophic neurites consistent with a primary neuropathologic diagnosis of FTLD-TDP-C. Co-pathologies included limbic stage Lewy Body Disease and low Alzheimer disease neuropathologic change [Thal: A1, Braak: B1 (Stage II), CERAD: C0], and severe arteriolosclerosis.

### FTLD-tau patient

The second patient was a 57-year-old, right-handed White woman with a nursing degree who presented to the Penn FTDC with a 2–3 year history of slowly progressive memory problems and behavioral disinhibition. She initially reported somatic complaints of sensory changes and déjà vu phenomena that were initially evaluated for epilepsy, but a workup was unrevealing, and she was referred for a cognitive evaluation.

The patient reported mild memory problems, but her family reported significant personality changes, including a new obsession with positivity and spirituality. She began to dress and dye her hair in bright colors and got a tattoo with a positive slogan which became a personal mantra. She exhibited disinhibition (e.g., approaching strangers with messages of positivity), impulsivity (e.g., attempting to sell her house suddenly without notifying her family), poor judgment (e.g., authoring a book on positivity that lost significant money to a predatory publisher), and reduced empathy (e.g., lacking appropriate concern following the death of a loved one). She became compulsive about creating artwork, including making frequent trips to buy large quantities of art supplies to make crafts that often conveyed positive messages with themes of spirituality (Figure 1C). She showed limited insight into these behavioral changes.

On examination, she had fluent speech and child-like affect. Confrontation naming was impaired despite relatively preserved object knowledge. Comprehension was intact, but she had surface dyslexia. Verbal memory showed retrieval-based impairment and visual recall was preserved. She had a poor mental search strategy, low letter fluency, and significant set-shifting difficulties. Despite problems with memory, executive functioning, and language, her MMSE was 30/30 at the initial visit. Elemental neurologic examination was unrevealing. Neuropsychological testing showed a pattern of prominent executive dysfunction with naming and semantic deficits and poor face recognition (Supplementary Table 2), and she met clinical criteria for bvFTD.

Within 4 years, her behavioral symptoms progressively worsened. She spent a large sum of money to buy a used car in poor condition despite having her driver’s license revoked, continued compulsively shopping and creating art, and obsessively recorded food and water intake. Her speech became more stereotypic (e.g., “Handsome boy brings me joy”), and she became agitated if ritualistic behaviors were disrupted. Her art and visual reproductions showed evidence of continued visual artistic creativity (Figure 1D); however, over time her artwork for animals were reported to show signs of degradation of semantic knowledge (e.g., turkey drawing with tentacles). Six years after symptom onset, she became dependent for self-care (CDR = 3, MMSE = 12) with significantly impaired comprehension (Supplementary Table 2), increasing apathy, and hyperorality with weight gain. She passed away with end-stage dementia 11 years after onset.

Neuropathologic examination found frequent tau pathology in the form of Pick bodies and ramified astrocytes, characteristic of PiD without co-pathology other than severe arteriosclerosis.

### Comparative antemortem MRI

**Figure 2:**
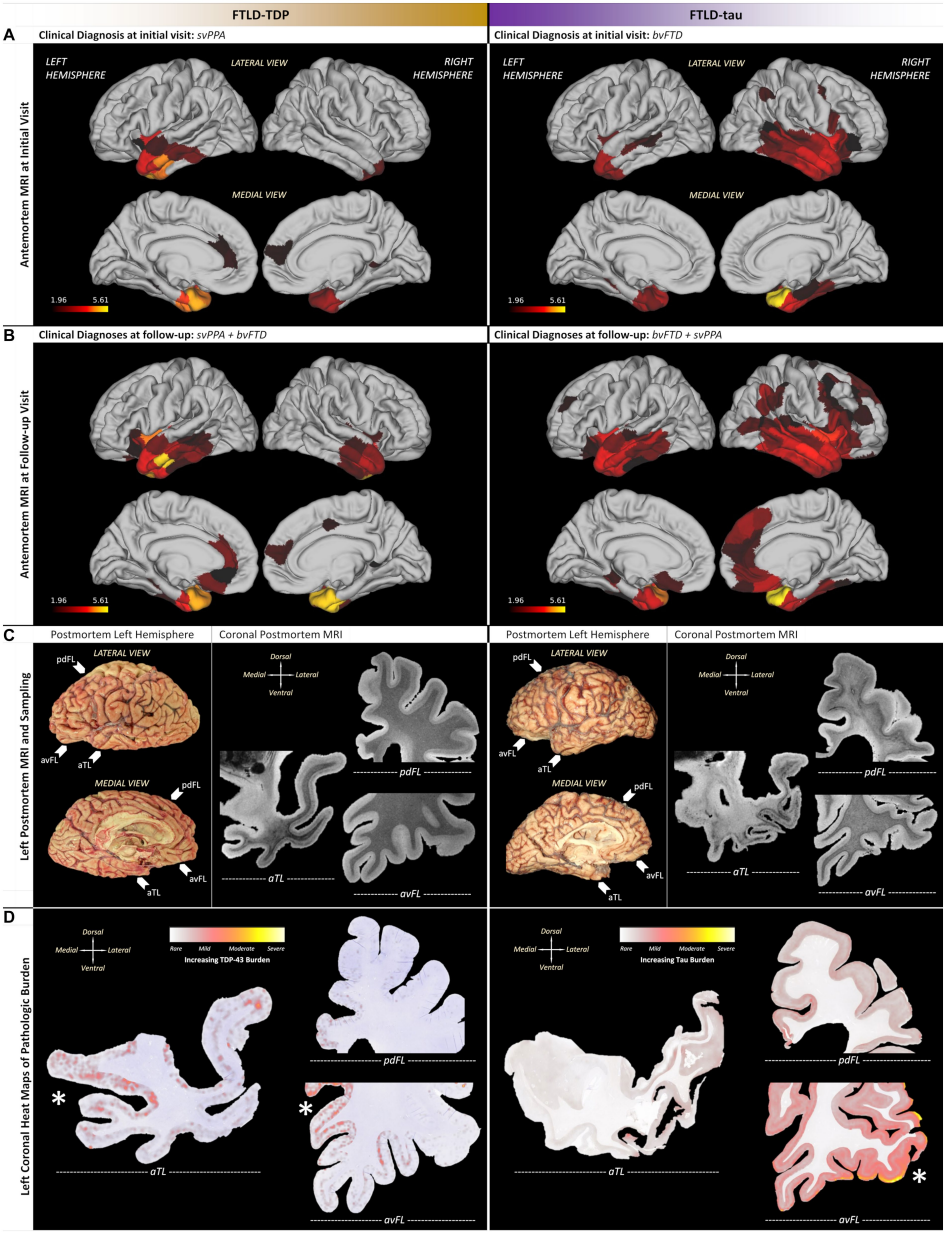
Patient-specific macroscopic patterns of cortical atrophy and neuropathologic changes using antemortem T_1_-weighted MRI and postmortem T_2_*-weighted MRI. **(A)** Initial MRI revealed a similar predilection for aTL but with opposing hemispheric asymmetry, including leftward asymmetry in the insula and anterior cingulate for FTLD-TDP, in contrast to rightward asymmetry in the insula and inferior frontal lobe for FTLD-tau. **(B)** Follow-up MRI in both patients showed spread and worsening of atrophy with preserved asymmetries observed in initial MRI, but the FTLD-tau patient showed greater spread in medial frontal and dorsolateral cortices throughout frontal, temporal, and parietal lobes. **(C)** In the left hemisphere of each patient, postmortem MRI helped identify aTL-predominant pathology and neurodegeneration that guided histopathology (arrows next to photographs of FIGURE 2 (Continued)autopsied brains point to whole hemisphere coronal sections used in subsequent comparative examinations of histopathology). **(D)** Quantitative heat maps of areas occupied by pathology identified patient-specific regions of peak pathology (denoted by asterisks near regions with greater red/yellow), including the FTLD-TDP patient accumulating greater TDP-43 burden in medial limbic/paralimbic regions with greater neurodegeneration, while the FTLD-tau patient accumulated greater tau burden in ventrolateral regions with relative neuronal preservation. Note that tau burden was consistently more frequent than TDP-43 burden; thus, scales of pathologic burden are distinct and specific to each molecular pathology due to normalization within each patient. aTL, anterior temporal lobe; pdFL, posterior dorsal frontal lobe; avFL, anterior ventral frontal lobe. All scale bars = 20 μm.

Baseline MRI within 2 years of symptom onset in each patient found a similar predominance of aTL atrophy in each patient, but with opposing hemispheric asymmetry (i.e., left > right in FTLD-TDP; right > left in FTLD-tau), consistent with initial clinical diagnoses (Figure 2A). Additionally, FTLD-TDP showed leftward asymmetry in the insula and anterior cingulate, whereas FTLD-tau showed rightward asymmetry in the insula and inferior frontal lobe.

Follow-up MRIs were acquired ~63% into clinical progression for the FTLD-TDP patient (i.e., 8 years after baseline MRI) vs. ~36% into clinical progression for the FTLD-tau patient (i.e., 2 years after baseline MRI) when both had mixed clinical features of bvFTD and svPPA (Figure 2B). In FTLD-TDP, cortical atrophy worsened but remained asymmetric in left frontotemporal regions with spread to left posterior orbitofrontal cortex, peri-genual cingulate cortices, and ventrolateral temporal cortices. In contrast, cortical atrophy remained rightward asymmetric in the FTLD-tau case and spread into the right medial frontal regions and dorsolateral cortices throughout frontal, temporal, and parietal lobes.

### Comparative postmortem MRI and histopathology

**Figure 3:**
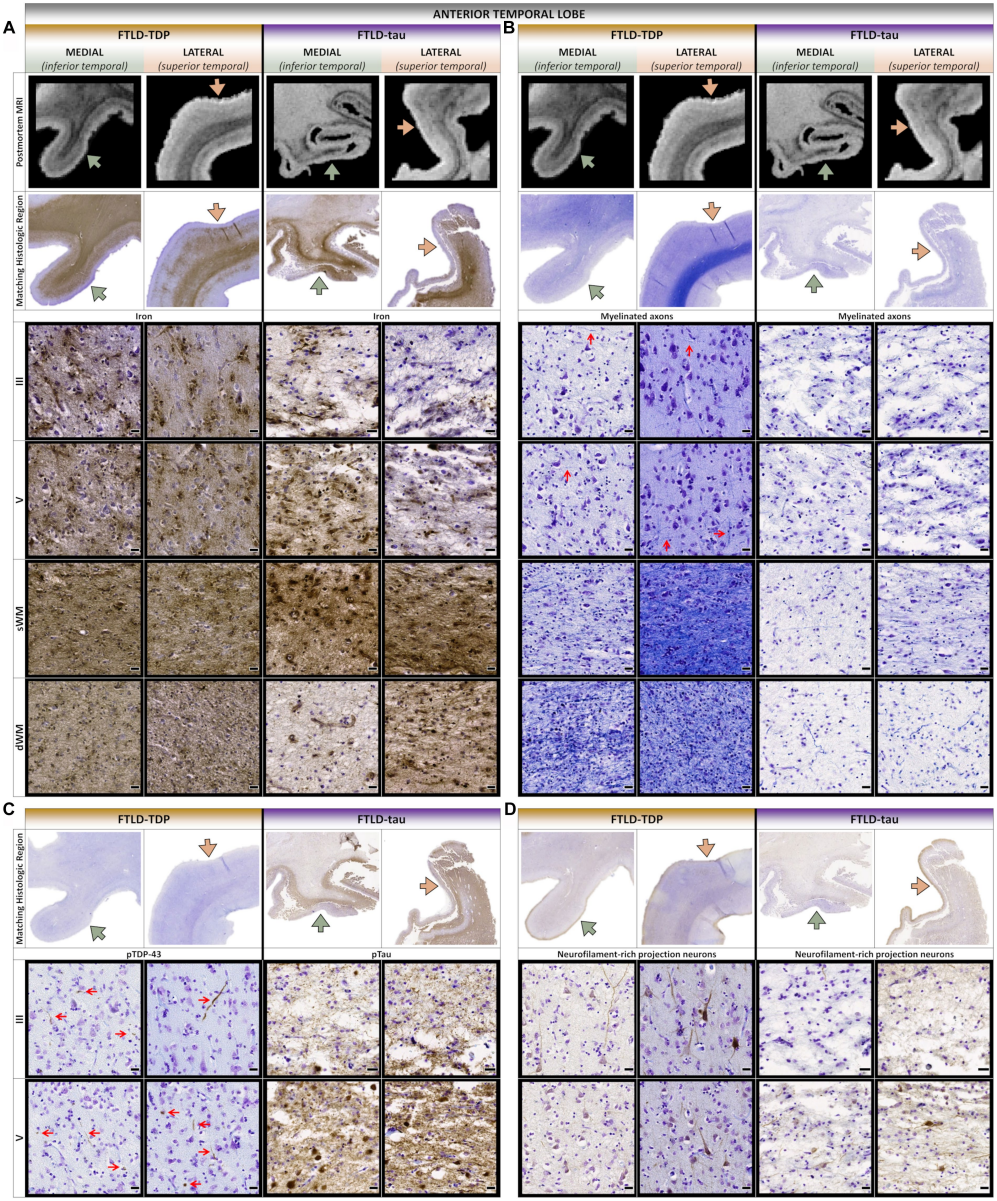
Comparative postmortem T_2_*-weighted MRI and histopathology of the anterior temporal lobe. **(A)** While iron frequently accumulated in lower layers and superficial white matter in both patients, the FTLD-TDP patient often displayed greater upper layer iron more concentrated to medioventral isocortices with greater atrophy and hypointensities. **(B)** Both patients showed severe loss of interlaminar and white matter myelin in medioventral isocortices, but the FTLD-tau patient showed more widespread and severe myelin loss, especially in dorsolateral isocortices and deep white matter. Red arrows point to examples of horizontally and vertically oriented myelinated fibers across cortical layers. **(C)** The FTLD-TDP patient accumulated TDP-43 inclusions in upper and lower layers throughout medial and lateral isocortices, but the FTLD-tau patient accumulated more tau inclusions in lower layers throughout medial and lateral isocortices. Red arrows point to examples of TDP-43 inclusions across cortical layers. **(D)** Both patients showed severe loss of projection neurons in upper and lower cortical layers, but the FTLD-tau patient displayed more severe neurodegeneration with more prominent vacuolation and neuropil disintegration. sWM, superficial white matter; dWM, deep white matter; all scale bars = 20 μm.

Postmortem T_2_*-weighted MRI contrast is sensitive to iron in healthy myelin (39) and iron-rich gliosis (40), revealing neurodegenerative patterns to guide histopathologic sampling (Figures 2, 3). Hypointensity from intracortical and deep myelin loss was most pronounced in the aTL (i.e., predominantly ventral temporal gyri) for both patients, but extended into dorsolateral frontal regions in PiD (Figure 2C). The FTLD-TDP patient displayed bands of upper layer hypointensity more concentrated to ventral isocortices, while the FTLD-tau patient showed variable laminar banding including mid and lower layer bands of hypointensity in more dorsolateral isocortices. These bands corresponded to iron-rich gliosis with hypertrophic, dystrophic, or amoeboid morphologies in both patients, including frequent iron-rich astrocytes near microvessels (Figure 3A).

To identify macro- (regional) and mesoscopic (laminar) patterns of histopathology, digital heat maps depicting the area occupied by pathologic inclusions found mild, patchy deposits of TDP-43 and tau pathology in the ventral aTL where atrophy was greatest (Figure 2D). In fact, the FTLD-TDP patient showed more (i.e., moderate-to-severe) TDP-43 pathology in regions with relatively less MRI-based atrophy than the ventral aTL, including the superior temporal gyrus, amygdala-hippocampal complex, peri-genual cingulate, and medial orbital gyri. Similarly, the FTLD-tau patient showed reduced tau burden in the severely atrophied aTL and the greatest tau burden in relatively less atrophied regions but in regions distinct from the FTLD-TDP patient, including pars orbitalis and Broca’s area.

Consistent with macro- and mesoscopic patterns reported above, microscopic patterns revealed a combination of shared and patient-specific laminar distributions of pathology and loss of projection neurons and axons. In the aTL, there was greater upper layer TDP-43 inclusions and loss of myelin and projection neurons in both upper and lower layers of medioventral gyri compared to relatively spared dorsolateral gyri in the FTLD-TDP patient (Figures 3B–D). In contrast, the FTLD-tau patient consistently showed severe tau pathology in layers II and IV-VI which corresponded to more severe and widespread loss of myelin and projection neurons in all layers and greater vacuolation/neuropil disintegration in lateral vs. medial regions. Moreover, deep white matter rarefaction and pathology was unique to the FTLD-tau patient and corresponded to hyperintense signal on postmortem MRI (Figures 3A–D).

**Figure 4:**
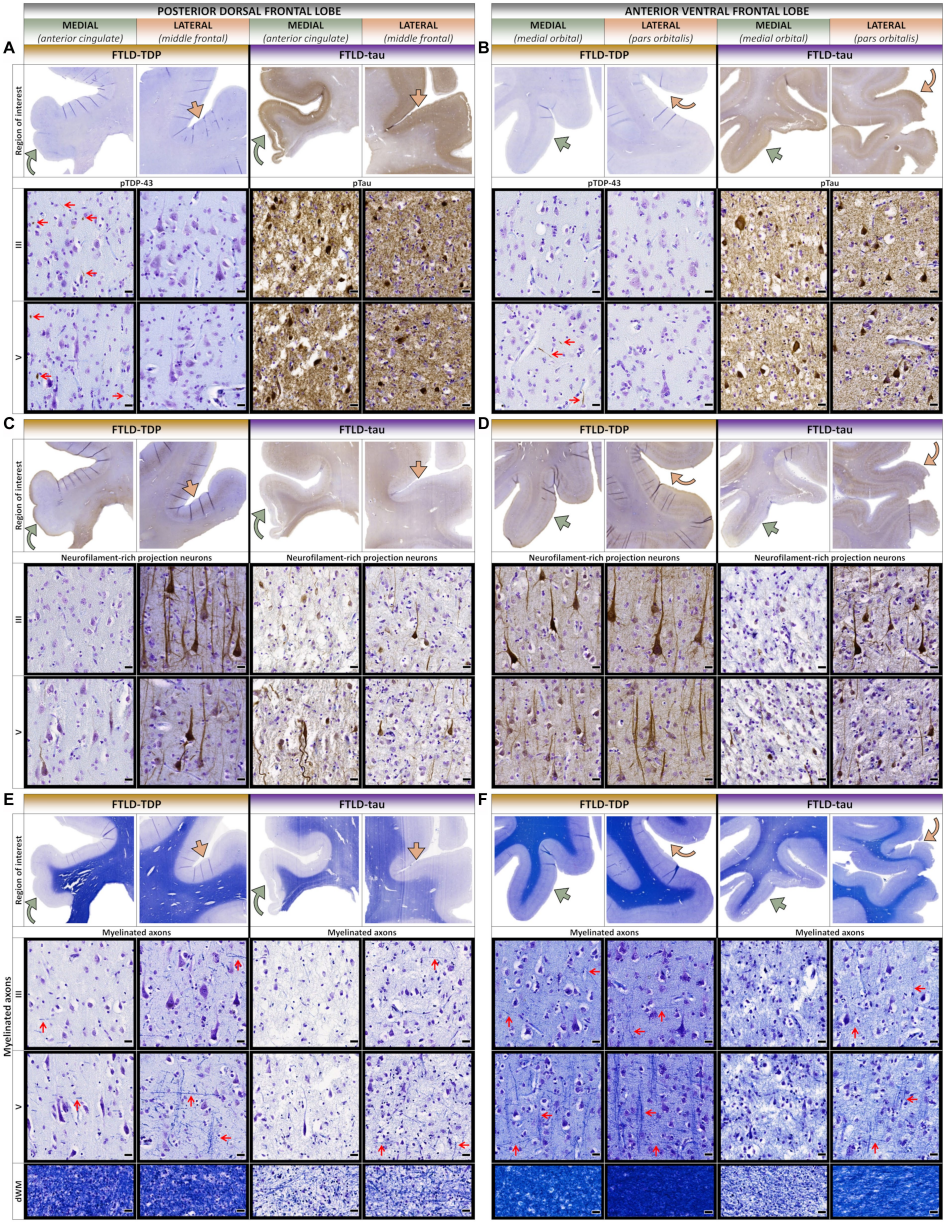
Comparative histopathology of the frontal lobe. **(A)** In the posterior dorsal frontal lobe (pdFL), the FTLD-TDP patient accumulated more TDP-43 pathology in upper and lower layers of medial proisocortical regions (e.g., anterior cingulate) compared to lateral isocortical regions (e.g., middle frontal). In contrast, the FTLD-tau patient accumulated severe pathology in both upper and lower layers consistently between medial and lateral regions. Red arrows point to examples of TDP-43 inclusions across cortical layers. **(B)** In the anterior ventral frontal lobe (avFL), the FTLD-TDP patient accumulated more TDP-43 pathology in predominantly lower layers of medial regions (e.g., medial orbital) compared to lateral regions (e.g., pars orbitalis). In contrast, the FTLD-tau patient accumulated severe pathology in both upper and lower layers consistently between medial and lateral regions. Red arrows point to examples of TDP-43 inclusions across cortical layers. **(C)** In the pdFL, the FTLD-TDP patient showed greater loss of FIGURE 4 (Continued)projection neurons in all layers of medial proisocortical regions compared to lateral isocortical regions. In contrast, the FTLD-tau patient showed a similar severe loss of projection neurons in all layers between medial and lateral regions. **(D)** In the avFL, the FTLD-TDP patient showed a slightly greater loss of projection neurons in all layers of medial regions compared to lateral regions. In contrast, the FTLD-tau patient showed greater loss of projection neurons in medial regions compared to lateral regions. **(E)** In the pdFL, the FTLD-TDP patient showed greater loss of interlaminar myelin in medial proisocortices, especially vertical myelinated fibers denoted by sideways-pointing red arrows compared to relatively preserved horizontal myelinated fibers denoted by red arrows pointing up. In contrast, the FTLD-tau patient showed overall greater myelin loss, particularly vertical myelinated fibers. Red arrows point to examples of horizontally and vertically oriented myelinated fibers across cortical layers. **(F)** In the avFL, the FTLD-TDP patient showed a slightly greater loss of myelin in medial regions compared to lateral regions, while the FTLD-tau patient showed more severe and widespread myelin loss between regions, with relatively rare preservation of myelinated fibers in lateral regions. Red arrows point to examples of horizontally and vertically oriented myelinated fibers across cortical layers. All scale bars = 20 μm.

In the frontal lobe, the FTLD-TDP patient accumulated more TDP-43 pathology in medial and ventral frontal regions (e.g., peri-genual cingulate and medial orbital gyri) comparable to the severity and layer involvement found in medioventral aTL (Figures 2D, 4A,B). Moreover, dorsal and lateral frontal regions with minimal neuron loss accumulated rare-to-mild TDP-43 inclusions in upper and lower layers. In contrast, medial and dorsolateral frontal regions of the FTLD-tau patient featured moderate-to-severe loss of projection neurons despite lower tau burden (Figures 4C,D). In both patients, loss of projection neurons and myelin were strongly concordant, especially the vertically oriented fibers traversing layers IV–VI (Figures 4E,F). However, the FTLD-tau patient displayed greater myelin loss in both gray and white matter, which corresponded to the overall greater loss of projection neurons and widespread tau pathology. In addition to myelin loss, the FTLD-tau patient displayed greater axonal loss most evident by the atrophied corpus callosum compared to the FTLD-TDP patient.

Comparison of available contralateral homologs found inconsistently greater TDP-43 pathology and neurodegeneration in left regions of the FTLD-TDP patient, while tau pathology and neurodegeneration were inconsistently greater in the right regions of the FTLD-tau patient (Supplementary Figure 1).

## Discussion

We present a detailed macro- and microanatomical investigation of two FTD patients with distinct FTLD proteinopathies that shared aTL-predominant atrophy associated with new onsets of compulsive and rigid belief systems. While their belief systems both centered on themes of positivity, the FTLD-TDP patient developed a complex schema of abstract concepts such as “intelligence and sensitivity” linked to pigs. In comparison, the FTLD-tau patient had a more generalized belief system of positivity with hyper-religious features that manifested in new artistic creativity. By pairing longitudinal clinical assessments with antemortem MRI and postmortem MRI-guided histology rarely collected in the same individuals, our multi-modal investigation revealed an early and shared susceptibility to aTL neurodegeneration concentrated to ventral aTL isocortices. However, divergent patterns of neurodegeneration between patients included disproportionate involvement of dorsal and lateral isocortex in the FTLD-tau patient, which corresponded to more prominent loss of projection neurons and long-range axons in infragranular layers and deep white matter. We hypothesize that early and severe degeneration of aTL and frontotemporal paralimbic regions contributed to semantic knowledge loss and the emergence of new belief systems. Moreover, we speculate that degradation of abstract concepts in the FTLD-tau patient was related to reduced integration of semantic concepts due to their severe frontoparietal degeneration compared to the FTLD-TDP patient. These data provide a unique view into the potential neural substrates of complex human value systems and FTD clinical symptomatology.

We found hemispheric and regional patterns of disease consistent with the initial clinical diagnoses of svPPA in the FTLD-TDP patient and bvFTD in the FTLD-tau patient (1, 10, 41). Initial MRI found left-predominant aTL atrophy in the FTLD-TDP patient and right-predominant frontotemporal atrophy in the FTLD-tau patient (Figure 2). Furthermore, these observations were confirmed postmortem in bilaterally sampled tissue (Supplementary Figure 1). The disproportionate involvement of the left aTL in the FTLD-TDP patient corresponds to the initial presentation of classic svPPA-related language symptoms (18–20, 41, 42). Accompanying these language changes were early behavioral features of dysphoria, impulsivity, and social disinhibition, which may be related to the patient’s additional atrophy of limbic and paralimbic hubs of the salience network, such as the frontoinsula and anterior cingulate (30, 43). In contrast, the FTLD-tau patient began with typical bvFTD symptoms including disinhibition, impulsivity, loss of empathy, and poor judgment consistent with their pronounced atrophy of right paralimbic regions (30, 44). However, there was also evidence for prosopagnosia and semantic language deficits that corresponded to the severe and extensive involvement of the bilateral temporal lobes. Her initial behavioral changes and subsequent language impairments are also largely consistent with the recently proposed criteria for the “semantic/right temporal” bvFTD phenotype (30). The FTLD-tau patient additionally had early somatic symptoms, hyper-religiosity, and episodic memory loss which are linked to right temporal disease (21, 23). In summary, both patients presented with a mix of language and behavioral features at the earliest clinical visits that were associated with early MRI-based atrophy of aTL and insula with more contralateral spread over time in both patients (Figure 2). Together our findings are consistent with previous literature (1, 16, 24) and suggest that hemispheric asymmetries of aTL disease likely influence the clinical features across the spectrum of bvFTD and svPPA.

Both patients also developed prominent complex rituals centered around their new belief systems that manifested in different ways. Specifically, the FTLD-TDP patient developed a new obsessive fixation on pigs early into disease course that eventually became the foundation of a broader belief system that supported an optimistic outlook and positive affect. Indeed, there was a temporal association between the onset of his pig obsession and improvement in mood symptoms that was stable until death (Supplementary Table 1). This new belief system featured new associations between concepts of intelligence and positivity with concrete anatomy or customs associated with pigs (Figure 1). The FTLD-tau patient developed a similarly intense fixation on positivity and happiness with spiritual overtones, but this manifested differently and more broadly by way of her artwork, personal appearance, and social behaviors. She also developed hoarding behavior in combination with rituals centered on shopping and daily production of visual artwork (Figure 1). The presence of complex rituals in both patients and artistic creativity in the FTLD-tau patient is consistent with data suggesting aTL atrophy contributes to complex goal-directed compulsive behavior (45, 46) and emergent visual creativity in FTD (33). Subcortical structures with dense connections to the aTL and regional hubs of the salience network have also been implicated in ritualistic and impulsive behavior in FTD, with evidence for basal ganglia involvement in aberrant reward processing (47) that may contribute to symptoms of excessive spending in the FTLD-tau patient and giving away money to strangers in the FTLD-TDP patient. These behaviors are consistent with existing reports of dissociable impairments in loss aversion (i.e., bias in decision making for avoidance of loss regardless of the potential gains or risk) and delayed discounting (i.e., perception of a greater value to immediate rewards than future rewards) in bvFTD and svPPA, respectively (48). Abnormal reward processing in FTD can resemble the impulse control phenomenon of reckless generosity previously described as a side effect of dopamine replacement therapy in Parkinson’s disease (49). However, unlike reckless generosity, which is due to hypersensitivity of the dopaminergic system and often reversible after reducing dopaminergic therapy in PD (50, 51), deficits in delayed discounting in svPPA appear driven by reduced valuation of future rewards leading and are linked to reduced connectivity of the anterior cingulate and medial temporal lobe (52) and atrophy in the dorsomedial prefrontal cortex, orbitofrontal cortex, subgenual anterior cingulate cortex, and ventral striatum that includes the dopaminergic system (53). Thus, a complex interaction of subcortical dopaminergic and cortical frontal and anterior temporal brain connectivity likely contributes to the observed symptoms of aberrant reward processing in FTD; however, the relatively selective degeneration of the aTL and paralimbic regions in the FTLD-TDP patient may have driven his impulsive generosity with strangers (54).

Differences in their new-onset belief systems may be explained in part by relatively unique anatomical involvement between patients. While semantic knowledge and social cognition are critically dependent on the integrity of the aTL, there are also contributions from connected regions. Indeed, a large body of literature supports a conceptual model in which bilateral aTL are neural hubs that integrate modality-specific inputs from distributed neocortical regions to generate generalizable semantic representations (55–58). Integration includes visual and auditory features from posterior temporal and occipital cortices, emotional valence from medial temporal and basal forebrain structures, and interoceptive cues from posterior frontal regions (55). In addition to the aTL, inferior parietal regions including the angular gyrus may contribute to semantic knowledge and integrative aspects of semantic processing to facilitate generalization of objects within a category (59–62). The FTLD-tau patient showed overall greater and more widespread neurodegeneration compared to the FTLD-TDP patient with a prominent loss of projection neurons and myelin/axonal loss associated with long-range tracts including severe atrophy of the corpus callosum and interlobar fiber bundles such as the uncinate and longitudinal fasciculi known to contribute to a multitude of language and behavioral impairments in FTD (63–66). In contrast, projection neuron loss and deep white matter changes were largely confined to the aTL in the FTLD-TDP patient. Moreover, these findings align with previous work demonstrating that white matter and projection neuron-rich lower layers accumulate severe tau pathology relatively spared by TDP-43 pathology (67, 68). We hypothesize these laminar distributions may also influence the more widespread spread of tau pathology to dorsolateral cortices across frontal, temporal, and parietal lobes typically associated with PiD (38, 69, 70) in contrast to the relatively focal involvement of left medial and ventral frontotemporal regions characteristic of TDP-C pathology (19, 31, 71, 72). One interpretation of these data is that the relatively focal left aTL disease in the FTLD-TDP patient may have led to a disconnection from temporoparietal areas and other integrative semantic hubs, resulting in inappropriate associations between concrete pig features (e.g., looking up) and less concrete concepts (e.g., positivity) relatively spared in svPPA (73). Despite the decline in semantic knowledge, the FTLD-TDP patient interestingly expressed the desire to communicate in numbers, possibly representing relative spared numeric comprehension subserved by posterior temporoparietal areas (59, 60, 74). He additionally showed improvement over time on several tests of executive functioning (i.e., Trail Making Test Part B, Letter Fluency) that may reflect relative sparing of prefrontal cortical regions and frontostriatal white matter connections that support executive processes (75). In contrast, the FTLD-tau patient developed a more generalized schema of positivity and had more diffuse cognitive impairment secondary to widespread disease across integrative hubs (76, 77).

Our ultra-high-resolution iron-sensitive MRI approach to guide histopathological sampling led to additional observations. While hypointense signal and iron-rich gliosis were predominantly found in the aTL of both patients, we found somewhat different laminar patterns. Similar to previous work (40, 78), select ventral aTL showed bands of upper layer hypointensity alongside variable mid and lower layer hypointense signal in the FTLD-TDP patient, while the FTLD-tau patient had more prominent but variable mid and deep layer bands of hypointensity in the aTL. These imaging findings corresponded to iron-rich gliosis, raising interesting questions about the potential role of non-cell autonomous neurodegeneration in FTLD. The localization of iron-rich gliosis to aTL epicenter regions may suggest this process occurs early in disease, or alternatively, is a later-occurring phenomenon associated with advanced neurodegeneration. PET imaging for microglial activation in living FTD patients suggests the former (79, 80), but further work is needed with autopsy confirmation.

The observed differences in macro-, meso-, and microscopic distributions of pathology between patients may suggest differences in the rate of neurodegeneration between proteinopathies and laminar patterns of cellular spread. Indeed, the FTLD-tau patient with 11 years of symptoms accumulated more severe and widespread pathology compared to the FTLD-TDP patient with a longer symptom course of 16 years. It is possible that the more extensive tau accumulation in all cortical layers for PiD (68, 69, 81–84) and concordant severe neurodegeneration of long-range projection neurons responsible for intra- and interhemispheric connections contributed to the more widespread neuropathologic changes in the FTLD-tau patient. In contrast, TDP-43 mediated neurodegeneration was relatively focal and most prominent in upper layers I-II consistent with previous work (19, 31, 68, 72, 85, 86), but unexpectedly frequent in additional layers V–VI. The cells bearing TDP-43-positive dystrophic neurites are currently understudied, but one possibility is that upper layer TDP-C pathology may originate from layer V/VI projection neurons whose distal ends of apical and basal dendrites are enriched in layers I–II and V–VI (87). The most severely degenerated regions of the aTL corresponded to relatively low accumulations of tau and TDP-43 pathology, consistent with previous findings in the aTL of patients with TDP-C or familial tauopathies (19, 72, 88). While TDP-43 burden and neurodegeneration were both greater medially than laterally in the FTLD-TDP patient, tau burden was higher in ventrolateral frontal isocortex with mild neurodegeneration compared to medial and dorsolateral frontotemporal cortices with peak neurodegeneration in the FTLD-tau patient. These patterns of partial overlap and divergence suggest a dynamic process of protein aggregation and neurodegeneration likely influenced by multiple factors, including regional-, cell-, and proteinopathy-specific mechanisms that contribute to variable rates of progression, duration, and clinical manifestations.

We find both convergent and divergent patterns of comparative neurodegeneration between FTLD proteinopathies that highlight potential mechanisms of selective vulnerabilities in FTD and neural substrates for complex human behavior. However, these observations will need to be confirmed in larger patient cohorts using methods integrating antemortem and postmortem neuroimaging with digital histopathology and antemortem behavioral assessments. While assessment of complex behavioral and neuropsychiatric symptoms in FTLD is methodologically challenging, advancements such as digital monitoring and event sampling using wearable devices and smartphones (e.g., activity tracking, psychophysiological monitoring) have the potential to capture more objective and ecologically valid metrics of behavioral disturbance (89, 90). These limitations notwithstanding, these illustrative descriptions can guide future large-scale, multi-modal studies of selective vulnerability in anatomic variants of FTD to further understanding of human values and belief systems.

## Data availability statement

The raw data supporting the conclusions of this article will be made available by the authors, without undue reservation.

## Ethics statement

The studies involving humans were approved by Institutional Review Board of the University of Pennsylvania. The studies were conducted in accordance with the local legislation and institutional requirements. Written informed consent for participation in this study was provided by the participants’ legal guardians/next of kin.

## Author contributions

DO, ER, and DJI contributed to the conception and design of the study. DO, ER, and DJI contributed to the first draft of the manuscript. All authors contributed to the article and approved the submitted version.

## Funding

This work was supported by NIH (grants NINDS R01-NS109260-01A1, NIA P01-AG066597, NIA P30-AG072979, NIA U01-AG052943-01, and NIA K01-AG-081484-01) and Penn Institute on Aging and DeCrane Family Foundation (K23-AG083124-01).

## Conflict of interest

The authors declare that the research was conducted in the absence of any commercial or financial relationships that could be construed as a potential conflict of interest.

## Publisher’s note

All claims expressed in this article are solely those of the authors and do not necessarily represent those of their affiliated organizations, or those of the publisher, the editors and the reviewers. Any product that may be evaluated in this article, or claim that may be made by its manufacturer, is not guaranteed or endorsed by the publisher.
