# Supplementary material for: Neuroanatomical and cellular degeneration associated with a social disorder characterized by new ritualistic belief systems in a TDP-C patient vs. a Pick patient
Source: Front Neurol. 2023 Oct 11;14:1245886. doi: 10.3389/fneur.2023.1245886 (PMC10600461; doi:10.3389/fneur.2023.1245886)
Supplement: Supplementary file 1 [file Data_Sheet_1.docx]

**Supplementary Material**

All procedures were performed with informed consent in accordance with Helsinki criteria and approved by the Penn Institutional Review Board.

**Methods (continued from main manuscript)**

***Neuroimaging***

Antemortem magnetic resonance imaging (MRI) was acquired with T_1_-weighted 3D MPRAGE sequences on a Siemens 3T scanner at Penn. Initial and follow-up MR scans were collected in each FTLD case and compared to a healthy control group comprising 159 neurologically normal participants. Data were processed using antsCorticalThickness **(**[**Tustison et al., 2014**](#_ENREF_76)**)** and collected from regions of interest (ROI) using the Schaefer 400x7 atlas **(**[**Schaefer et al., 2017**](#_ENREF_70)**)**. Cortical atrophy was derived from “W-scores” calculated in each ROI volume by using linear regression to remove age and sex effects based on control scans. Values below -1.96 were considered significant (p<0.05) cortical atrophy and visualized as cortical heat maps (**Fig. 2**).

Postmortem T2*-weighted MRI was performed in only the left hemisphere of each patient following procedures reported previously **(**[**Tisdall et al., 2022**](#_ENREF_75)**)**. After autopsy, whole hemispheres of both patients were submerged in 10 % Neutral-buffered formalin for >60 days prior to MRI scanning (FTLD-TDP= 241 days; FTLD-tau=108 days). Within 48 hours after autopsy and before postmortem MRI, 14 regions relevant to FTD were sampled from the intact left hemisphere of the FTLD-tau patient to compare to the contralateral (right) hemisphere sampled for neuropathologic diagnostics (see details below). The left hemisphere of the FTLD-TDP patient remained intact before postmortem MRI, and we compared our postmortem MRI-guided histopathology in the left hemisphere to contralateral homologues collected at autopsy for neuropathologic diagnosis.

Each left hemisphere was packed into custom-built 3D-printed holders within a bag containing proton-free fluid with volume magnetic susceptibility close to that of tissue (i.e., Fomblin; California Vacuum Technology; Freemont, CA). MRI data were acquired with a Siemens 7T scanner at Penn using a 3D-encoded, 8-echo gradient-recalled echo (GRE) sequence with non-selective RF pulses with the sequence: 160 μm isotropic resolution, 25◦ flip angle, 60 ms repetition time (TR), 9.37 ms minimum echo time (TE), 11.33 ms echo spacing, and 90 Hz/px bandwidth similar to methods previously described **(**[**Tisdall et al., 2022**](#_ENREF_75)**)**.

***Digital Histopathology***

Diagnostic tissue from 20 regions relevant to FTD were uniformly collected, formalin-fixed, paraffin-embedded, and sectioned at 6 µm-thickness from the right hemisphere of both patients. In the FTLD-tau patient only, 14 contralateral homologues were sampled from the intact left hemisphere and sectioned at 6 µm-thickness before postmortem MRI.

The postmortem MRI was used to construct a 3D-printed mold for each hemisphere to guide coronal slabbing. The left hemisphere of both patients was cut into 1 cm-thick, whole-hemisphere coronal blocks and bisected or trisected along the axial plane to fit 2x3-inch cassettes and processed for paraffin-embedding. Coronal blocks were sectioned at 20 µm-thickness. Semi-adjacent sections were histochemically or immunohistochemically stained to evaluate the distribution and severity of neurodegenerative changes, including hyperphosphorylated tau using AT8 (1:1000, Invitrogen), and phosphorylated TDP-43 using TDP-43 (1:1000, ProteinTech), myelin using Luxol Fast Blue using Kluver-Barra method (0.1% Luxol Fast Blue in 95% ethanol, Acros Organics), iron using a modified Meguro method **(**[**Duijn et al., 2013**](#_ENREF_11)**)**, and neurofilament-rich projection neurons using SMI-32 (1:300, BioLegend). All sections were counterstained with Nissl cresyl violet (0.1% cresyl violet in distilled water) to identify cortical layers and provide further assessment of gliosis and neurodegeneration. Whole-slide images (WSI) were obtained using a Huron Tissue Scope LE120 (Huron Digital Pathology, St. Jacobs ON).

Heat maps of histopathologic severity from WSI were generated using 20 µm-thick left hemisphere coronal sections immunohistochemically stained for TDP-43 in the FTLD-TDP patient or tau pathology in the FTLD-tau patient. First, a trained anatomist (DTO) annotated the entire cortical ribbon available on each tissue section using QuPath software. Next, the WSI was exported as a json file to overlay a grid of tiles (1024x1024 pixels each) such that only pixels inside of the cortical ribbon annotation were included in analysis. To detect immunoreactive pathologic inclusions, an adaptive thresholding algorithm was applied to each tile to first remove non-specific background by subtracting the average signal surrounding each pixel, and then setting a threshold using a maximum deviation thresholding algorithm. The thresholding algorithm uses the histogram from the non-specific background signal and an elbow method to select the optimal threshold per tile. Finally, all thresholded tiles are stitched together and a gaussian kernel is convolved over the WSI to produce a continuous cortical heat map. Due to signal intensity variation between sections and FTLD brains, signal intensity was normalized based on the maximum percent area occupied (%AO) measured from all three sections within each FTLD patient as follows:

[normalized %AO] = [raw %AO] / [maximum %AO per patient across three sections]

Given that tau pathology reached a higher magnitude than TDP-43 pathology, the scale normalization results in relative magnitudes of pathologic burden specific to each molecular pathology. Heat maps are overlaid to downsampled images of the original immunostained tissue sections to reliably render visualizations of the relative distributions of pathologic burden of each molecular pathology for direct comparison of anatomical patterns at mesoscopic (laminar) and macroscopic (regional) resolutions (**see Fig. 2D**).

**
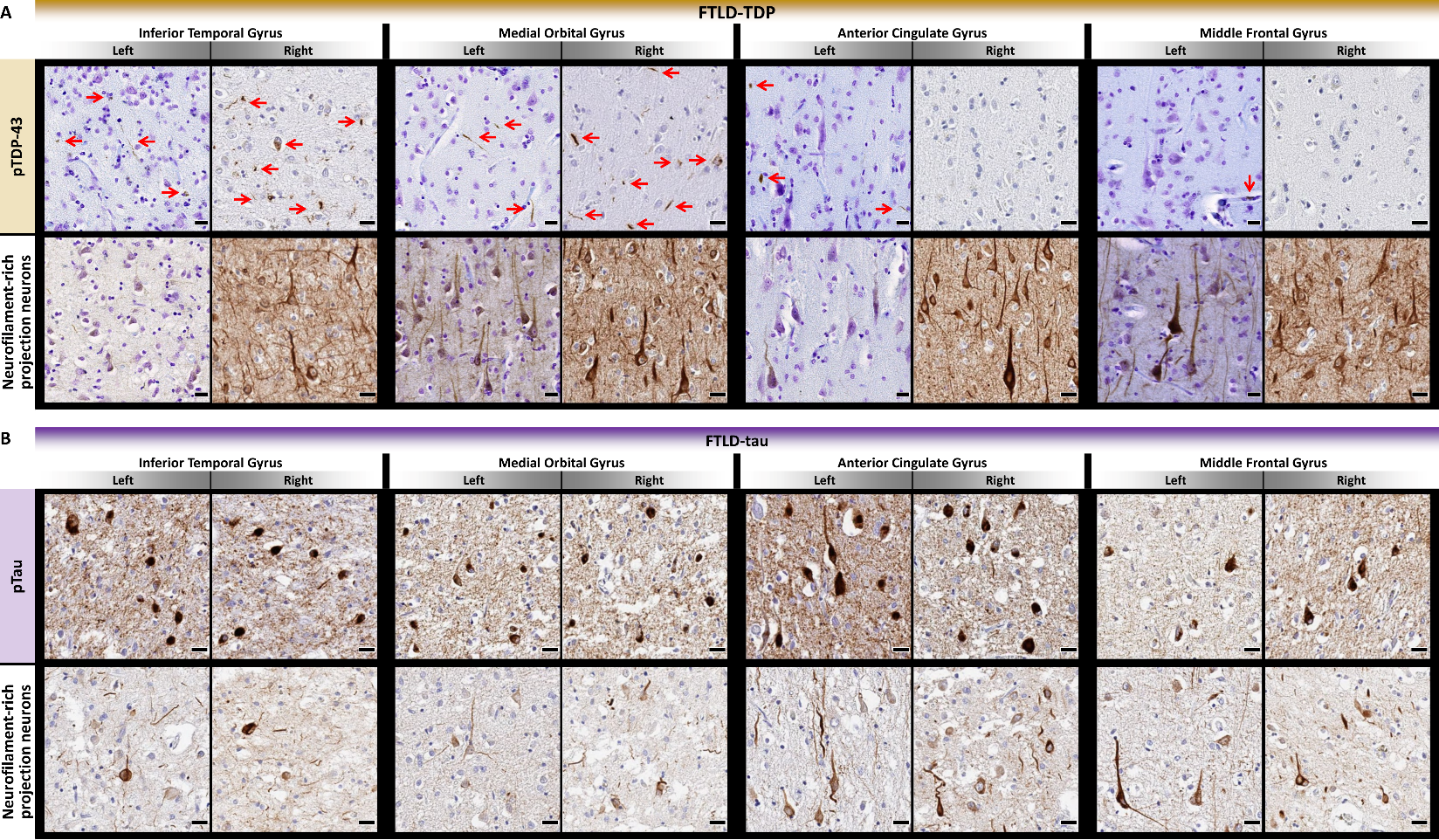
Supplementary Figure 1. Bilateral comparison of pathology and neurodegeneration in each FTLD patient.**

**A)** In the FTLD-TDP patient, we inconsistently found more severe TDP-43 pathology and neurodegeneration in left regions. Red arrows point to examples of TDP-43 inclusions across cortical layers. Note that left regions were examined in 20 µm-thick tissue and right regions were examined in 6 µm-thick tissue.

**B)** In the FTLD-tau patient, we found tau pathology and neurodegeneration were often similar between hemispheres and occasionally greater in the right hemisphere. Bilateral regions examined in equivalent 6 µm tissue.

All photomicrographs acquired in layer V; all scale bars = 20 µm

**Supplementary Table 1. Longitudinal neuropsychological testing data for the FTLD-TDP patient.**

| **Years Following Diagnosis** | **2** | **8** | **10** | **12** |
| --- | --- | --- | --- | --- |
| **NACC UDS-3** |  |  |  |  |
| MMSE/MoCA Total | 24^†^ | 18^†^ | 14 | 12 |
| Craft Story Immediate Recall - Verbatim | - | - | 5 | 8 |
| Craft Story Immediate Recall - Paraphrase | - | 1 | 3 | 6 |
| Benson Copy | - | 15 | 17 | 14 |
| Number Span - Forward | - | 5 | 5 | 4 |
| Number Span - Backward | - | 4 | 3 | 2 |
| Category Fluency - Animals | - | 9 | 6 | 6 |
| Category Fluency - Vegetables | - | 1 | 1 | 1 |
| Trail Making Test - A | - | 42 | 45 | 40 |
| Trail Making Test - B | - | 300 | 135 | 181 |
| Craft Story Delayed Recall - Verbatim | - | - | 0 | 4 |
| Craft Story Delayed Recall - Paraphrase | - | 0 | 0 | 4 |
| Benson Recall | - | 10 | 8 | 10 |
| BNT/MINT | - | 4 | 7 | 3 |
| Letter Fluency - F | - | 7 | 7 | 8 |
| Letter Fluency - L | - | 8 | 10 | 14 |
| **FTLD CERAD** |  |  |  |  |
| Word Reading - Regular | 5 | 9 | 13 | 10 |
| Word Reading - Irregular | 10 | 6 | 2 | 4 |
| Semantic Word-Picture Matching | 20 | 15 | 17 | 17 |
| Semantic Associates Total | 12 | 11 | 13 | 11 |
| Northwestern Anagram | 9 | 8 | 6 | 8 |
| Sentence Repetition | 1 | 2 | 2 | 3 |
| Noun Naming | 8 | 7 | 8 | 7 |
| Verb Naming | 10 | 13 | 12 | 12 |
| Sentence Reading | 3 | 3 | 2 | 1 |
| **Pyramids and Palm Trees** |  |  |  |  |
| Pictures | 47 | 21 | 20 | 20 |
| Words | 39 | 17 | 17 | 13 |
| **Neuropsychiatric Inventory - Severity Ratings** |  |  |  |  |
| Delusions | 0 | 0 | 0 | 0 |
| Hallucinations | 1 | 0 | 0 | 0 |
| Agitation | 0 | 2 | 0 | 0 |
| Depression/Dysphoria | 1 | 0 | 0 | 0 |
| Anxiety | 1 | 2 | 1 | 1 |
| Euphoria/Elation | 1 | 3 | 1 | 2 |
| Apathy/Indifference | 2 | 2 | 2 | 2 |
| Disinhibition | 1 | 3 | 1 | 2 |
| Irritability/Lability | 1 | 2 | 1 | 1 |
| Aberrant Motor Behavior | 0 | 3 | 1 | 1 |
| Sleep/Night Time Behavior | 3 | 1 | 1 | 1 |
| Appetite/Eating | 3 | 3 | 2 | 1 |
| Total Severity Score | 14 | 21 | 10 | 11 |

*UDS-2; ^†^MMSE

**Supplementary Table 2. Longitudinal neuropsychological testing data for the FTLD-tau patient.**

| **Years Following Diagnosis** | **3** | **5** | **6** |
| --- | --- | --- | --- |
| **NACC Data** |  |  |  |
| MMSE/MoCA Total | 24^†^ | 7 | 4 |
| Craft Story Immediate Recall - Verbatim | 5 | 4 | 1 |
| Craft Story Immediate Recall - Paraphrase | - | 0 | 1 |
| Benson Copy | 17 | 16 | 16 |
| Number Span - Forward | 7 | 6 | 6 |
| sNumber Span - Backward | 7 | 5 | 3 |
| Category Fluency - Animals | 11 | 7 | 5 |
| Category Fluency - Vegetables | 5 | 0 | 0 |
| Trail Making Test - A | 85 | 111 | 69 |
| Trail Making Test - B | 126 | - | - |
| Craft Story Delayed Recall - Verbatim | 1 | 0 | 0 |
| Craft Story Delayed Recall - Paraphrase | - | 0 | 0 |
| Benson Recall | 0 | 6 | 0 |
| BNT/MINT | 14 | 0 | 2 |
| Letter Fluency - F | 7 | 5 | 5 |
| Letter Fluency - L | 6 | 4 | 2 |
| **FTLD CERAD** |  |  |  |
| Word Reading - Regular | 15 | 15 | 12 |
| Word Reading - Irregular | 13 | 9 | 3 |
| Semantic Word-Picture Matching | 19 | 17 | 14 |
| Semantic Associates Total | 15 | 9 | 2 |
| Northwestern Anagram | 8 | - | - |
| Sentence Repetition | 5 | 5 | 4 |
| Noun Naming | 14 | 3 | 3 |
| Verb Naming | 15 | 6 | 3 |
| Sentence Reading | 5 | 5 | 5 |
| **Pyramids and Palm Trees** |  |  |  |
| Pictures | 18 | 18 | - |
| Words | 11 | 10 | - |
| **Neuropsychiatric Inventory - Severity Ratings** |  | - | - |
| Delusions | 0 | - | - |
| Hallucinations | 3 | - | - |
| Agitation | 0 | - | - |
| Depression/Dysphoria | 0 | - | - |
| Anxiety | 3 | - | - |
| Euphoria/Elation | 2 | - | - |
| Apathy/Indifference | 2 | - | - |
| Disinhibition | 0 | - | - |
| Irritability/Lability | 2 | - | - |
| Aberrant Motor Behavior | 1 | - | - |
| Sleep/Night Time Behavior | 0 | - | - |
| Appetite/Eating | 0 | - | - |
| Total Severity Score | 13 | - | - |

*UDS-2; ^†^MMSE
